# Healthcare workers’ perspectives and practices regarding the disclosure of HIV status to children in Malawi: a cross-sectional study

**DOI:** 10.1186/s12913-018-3354-9

**Published:** 2018-07-11

**Authors:** Fatch W. Kalembo, Garth E. Kendall, Mohammed Ali, Angela F. Chimwaza

**Affiliations:** 1 School of Nursing, Midwifery and Paramedicine, Curtin University, Perth, Australia; 2 Mzuzu University, Mzuzu, Malawi; 3 Kamuzu College of Nursing, University of Malawi, Blantyre, Malawi

**Keywords:** HIV status, Disclosure, Practice, Barriers, Healthcare workers, Children

## Abstract

**Background:**

In 2011 the World Health Organisation recommended that children with a diagnosis of HIV be gradually informed about their HIV status between the ages of 6 and 12 years. However, to date, literature has focused mainly on primary caregiver and child experiences with HIV disclosure, little is known about healthcare workers’ perspectives and practices of HIV status disclosure to children. The aim of this study was to assess healthcare workers’ perspectives and practices regarding the disclosure of HIV status to children aged between 6 and 12 years in Malawi.

**Methods:**

A cross-sectional survey was used to collect data from 168 healthcare providers working in antiretroviral clinics in all government District and Tertiary Hospitals in Malawi. Participants were asked questions regarding their knowledge, practice, and barriers to HIV disclosure. Data were analysed using binary logistic regression.

**Results:**

Almost all healthcare workers (98%) reported that it was important to disclose HIV status to children. A significant proportion (37%) reported that they had never disclosed HIV status to a child and about half estimated that the rate of HIV disclosure at their facility was 25% or less. The main barriers to disclosure were lack of training on disclosure (85%) and lack of a standard tool for disclosure (84%). Female healthcare workers (aOR) 2.4; 95% CI: 1.1–5.5) and lack of training on disclosure (aOR 7.7; 95% CI: 3.4–10.7) were independently associated with never having disclosed HIV status to a child.

**Conclusions:**

This study highlights the need for providing appropriate training in HIV disclosure for healthcare workers and the provision of standardised disclosure materials.

**Electronic supplementary material:**

The online version of this article (10.1186/s12913-018-3354-9) contains supplementary material, which is available to authorized users.

## Background

Mother-to-child infection during childbirth and lactation has been the major mode of transmission of HIV in children globally since the first case of HIV was identified in the 1980s [1]. During the 1980s and early 1990s, many children living with HIV died before their fifth birthday [2]. The introduction of antiretroviral (ART) medications in the 1990s dramatically increased longevity, by 10 or more years, and brought hope to affected children, their family members, and healthcare workers [3]. As a consequence of many children surviving into adolescence and even early adulthood, new issues have arisen about their health and wellbeing, as well as the potential risk to others associated with sexual contact [4]. A crucial question is the appropriate age at which children should be told about their HIV diagnosis [3]. Disclosure remains a significant issue, especially in sub-Saharan Africa, despite the World Health Organisation (WHO) publishing evidence-based guidelines that recommend children be given developmentally-appropriate information about their HIV status gradually from the age of six through to 12 years [5].

Non-disclosure has been associated with lack of ART medication adherence, which can lead to the development of drug-resistant strains [6–8] and the potential for children to unknowingly transmit the virus to others once they reach adolescence [6, 9]. On the other hand, disclosure of HIV status has been associated with stigma and discrimination being directed at the family [10–14] and concerns about the child’s ability to cope with the diagnosis [6, 9].

Despite the WHO recommendations and guidelines for disclosure, recent studies reveal that the rate of disclosure of HIV status to children in the sub-Saharan region remains low [15–17]. The disclosure rate identified in studies in South Africa, Zambia Tanzania, Kenya, and Ethiopia have ranged from 11 to 34% only [15–20]. While the WHO guidelines recommend that disclosure of HIV status to children can be carried out by any person acting in the best interests of the child [5], most of the published literature endorse the primary caregiver as the person with the principal responsibility to disclose to their child [6, 8, 21–23]. However, recent studies have revealed that many primary caregivers find the disclosure process difficult and require considerable assistance from healthcare workers [24–26].

Healthcare workers are potentially central to the disclosure process because they provide medical and psychosocial care to both children living with HIV and their families [22, 23, 27]. Moreover, they share the secret of the child’s HIV status with the primary caregiver [18]. Due to the stigma associated with the child’s HIV diagnosis, many parents are reluctant to discuss their concerns within the community and seek psychosocial support [28]. As such, healthcare workers are in the best position to assist the primary caregiver in decision-making regarding when, how and who is the most appropriate person to disclose to their child [23, 27]. Nonetheless, studies have shown that many healthcare workers are not actively involved in the disclosure process [29, 30]. A lack of professionalism and empathy among healthcare workers was reported to hinder primary caregivers disclosing to their child in a study conducted in Kenya [30]. A South African study reported complaints from primary caregivers of rude and uncaring behaviour among some healthcare workers when they came with their children to receive care [29]. On the other hand, studies that have assessed healthcare workers’ experiences of disclosure of HIV to children in sub-Saharan Africa have revealed that a lack of training and skills about the disclosure process [13, 31], lack of cooperation from primary caregivers, lack of time [32, 33], and lack of standardised disclosure policies and materials [13, 31] are the major impediments.

In 2015, there were 84,000 children under the age of 14 years (1.6% of the total population) living with HIV in Malawi [34]. Of these, 60% were prescribed ART [34]. With the widespread provision of ART, children with HIV are living substantially longer than before [35]. It is expected that most children living with HIV in Malawi and other sub-Saharan countries will in time achieve an average lifespan, similar to that achieved today in high-income countries [36]. It is critically important that children living with HIV know that they have the disease and that they are able to manage it properly so they maximise their own health and wellbeing and minimise the threat they pose to others [37].

In Malawi, there is very little data on the current practice of HIV disclosure to children living with HIV. Moreover, to the best of the authors’ knowledge, no study in Malawi has assessed healthcare workers’ involvement in the disclosure of HIV status to children. It is thus timely to assess these perspectives. Given the significant role they play in caring for children living with HIV, such a study would help inform the development of effective professional practice guidelines. The aim of this study was to assess healthcare workers’ perspectives and practices regarding the disclosure of HIV status to children aged between 6 and 12 years in Malawi. Specific research questions were: 1. What is the proportion of healthcare workers who have never disclosed; 2. What are the perceived barriers to non-disclosure; and 3. What are the sociodemographic and professional characteristics of healthcare workers who have never disclosed.

## Methods

### Study design and setting

A cross-sectional study of healthcare professionals working in ART clinics in all government District and Central (Tertiary) Hospitals in Malawi was carried out during March–July, 2015. Of the 28 districts in Malawi, 23 have government District Hospitals. The five districts with no government District Hospital either rely on government Central Hospitals or Christian Association of Malawi Hospitals for services. There are four government Central Hospitals in Malawi available to provide ART services. However, only three Central Hospitals (Mzuzu, Zomba and Queen Elizabeth Central Hospitals) were included in data collection. The remaining Central Hospital (Kamuzu Central Hospital) was excluded because it did not have an ART clinic and instead relied on two privately run ART clinics (Baylor College and Lighthouse clinics) for ART services. Each Central or District Hospital has an HIV department known as antiretroviral therapy (ART) clinic where people living with HIV including children receive their ART medication and other related HIV care services. The ART clinics are designated areas in the hospitals where data collection took place.

### Recruitment of study participants and sampling

To participate in the study, potential participants were required to meet the following eligibility criteria: (i) working in the ART clinic, (ii) being a nurse, clinical officer/doctor or counsellor, and (iii) providing consent to participate in the study. While not an exclusion criterion, it was anticipated that all participants would be conversant in English. All healthcare professionals working in ART clinics in government District and Central Hospitals in Malawi were approached to participate in the study and those who consented were recruited. Normally, each ART clinic has a minimum of three healthcare workers (a clinical officer, a nurse (registered nurse or nurse technician), and a counsellor/clerk).

In Malawi, registered nurses undergo three to 4 years of university training in nursing before qualifying with a Diploma or Degree in Nursing. With regard to ART care, their duties include: provision of drugs, providing counselling to new patients, and assessing patients for drug compliance and adherence. Nurse technicians are a group of nurses who undergo a 3 years nursing training at a nursing college that is not affiliated with a university. They qualify with a College Diploma in Nursing and Midwifery. Their duties in the ART clinic are similar to those of a registered nurse, however, they do not have a supervisory role.

The clinical officers (clinicians) receive 4 years full training in clinical medicine including supervised clinical practice and internship and they qualify with a Diploma in Clinical Medicine. Due to the critical shortage of medical doctors in Malawi, clinical officers work as medical officers. Their main duties in the ART clinic are assessing patients for ART eligibility and commencing them on treatment. Another group of healthcare workers assigned to the ART clinic are counsellors. These are health workers who were previously working as health surveillance assistants, patient attendants or data clerks. They undergo an in-service training on counselling. Their duties in the ART clinic include the provision of pre- and post-HIV testing counselling as well as testing people who want to know their HIV status.

### Data collection procedure

District Nursing Officers and Nursing Officers were chosen as focal people to assist in recruiting participants and distributing and collecting questionnaires. District Nursing Officers are registered nurses who are in-charge of nurses in an entire district. While Nursing Officers are registered nurses who are in-charge of a ward or department at a secondary or tertiary hospital. Orientation sessions were held to inform them about the purpose of the study and the data collection procedure. Questionnaires, information sheets, and consent forms, all in English, were mailed to them, along with a stamped return envelope. The focal people approached the health professionals in their respective health facilities who met the recruitment criteria. Those who consented to participate in the study were given the questionnaire to complete with instructions on how to complete it. Completed questionnaires were returned to the focal people who subsequently sent them to the research team by post. Participants were instructed not to write their names on the completed questionnaires, as it was important that responses remained anonymous. Ethical approval to conduct the study was obtained from Curtin University Human Ethics Committee and the Malawi Government Health Science Research Committee prior to the commencement of data collection. Permission to collect data was also obtained from the District Health Officers and the Directors of the hospitals where data collection was conducted.

### Data collection instrument

The study questionnaire was developed by the authors who were guided by the research questions and informed by literature regarding the disclosure of HIV status to children [22, 23, 38–40]. The questionnaire had two sections: a) socio-demographic information of the participants and b) current practice and challenges related to HIV disclosure to children. The full questionnaire is presented as Additional file 1. The instrument was piloted with healthcare workers at a healthcare facility which was excluded from the study sample.

### Statistical analysis

Descriptive statistics of healthcare worker’s demographic characteristics and knowledge, challenges, and practices of disclosure were tabulated. Bivariate analysis was conducted using logistic regression in order to find the odds of all independent variables being associated with non-disclosure among the healthcare workers. The outcome variable of the study was HIV non-disclosure. The independent variables were: age, gender, work experience in ART, ART training, and professional group. These variables were selected following our review of previous studies [22, 23, 38–40] whose authors reported on healthcare workers’ perspectives on HIV disclosure. Variables with *p*-values of ≤0.25 in bivariate analysis [41] were then included in the multivariate logistic regression in order to find adjusted odds ratio of variables associated with non-disclosure The level of significance was set at *p* < 0.05.

## Results

A total of 175 healthcare providers working in all public District and Central Hospitals ART clinics in Malawi were approached to participate in the study. Two refused to participate, and three did not meet eligibility criteria. A total of 170 questionnaires were collected representing a response rate of 99%. Two questionnaires were excluded from data analysis because they had many unanswered questions. A total of 168 questionnaires were included in the data analysis.

### Socio-demographic characteristics and HIV disclosure practices

Descriptive statistics of healthcare worker socio-demographic characteristics and their HIV disclosure practices are presented in Table 1. Most participants (91%) were from District hospitals. Age was evenly distributed between the three categories (21–30, 31–40, > 40 years). Nurse technicians were the largest professional group (34%), followed by counsellors (29%), clinical officers (23%), and registered nurses (14%). The majority of healthcare workers (76%) had more than 2 years of working experience in ART clinic. All regions of Malawi were well represented; however, the highest proportion (48%) of healthcare workers came from the Southern region which is the most populated in the country. Almost equal numbers of males and females responded to the survey.

**Table 1:** Healthcare workers’ socio-demographic characteristics and disclosure of HIV status to children (*N* = 168)

Characteristic	n (%)
Level of health facility	
District hospital	153 (91)
Central hospital	15 (9)
Age	
21–30	60 (36)
31–40	57(34)
> 40 years	51(30)
Profession	
Nurse technician	56 (34)
Registered nurse	24 (14)
Counsellor	49 (29)
Clinical officer	39 (23)
Years of working in ART clinic	
0–1	40 (24)
2–3	67 (40)
≥ 4 years	61(36)
Region	
Central	48 (29)
North	39 (23)
South	81 (48)
Gender	
Female	86 (51)
Male	82 (49)
Disclosure of HIV status to children	
Necessary to disclose to children	
Yes	165 (98)
No	3 (2)
Ever disclosed HIV to a child	
Yes	106 (63)
No	62 (37)
Prepared the child prior to disclosure	
Yes	102 (96)
No	4 (4)
Provided follow-up care to the child after disclosure	
Yes	94 (89)
No	12 (11)
Duration of the disclosure process	
One occasion only	15 (14)
2–3 occasions	33 (31)
4–6 occasions	6 (6)
Over 6 occasions	52 (49)
Best age for HIV disclosure	
< 6 years	12 (7)
6–12 years	101 (60)
≥ 13 years	51 (31)
I don’t know	4 (2)
Best person to disclose HIV to a child	
Primary caregiver	37 (22)
Healthcare worker	37 (22)
Primary caregiver and healthcare worker	94 (56)
Rate of HIV disclosure to children at the facility	
0–25%	86 (51)
26–55%	42 (25)
Above 55%	40 (24)
Received in-service disclosure training	
Yes	79 (47)
No	89 (53)

Ninety-eight per cent of the participants reported that they believed it was necessary to disclose HIV status to children living with HIV. Despite this belief, 37% of healthcare workers reported that they had never disclosed HIV status to a child. For those who reported having ever disclosed, 96% reported that they had prepared the child prior to disclosure by telling them about the causes and transmission of the disease. Almost half (49%) reported that disclosure took place gradually over six or more occasions. Sixty per cent identified 6 to 12 years as the best age range in which to disclose HIV to children, while 31% identified 13 years and over to be the best age. Twenty-two per cent of healthcare workers thought that they were best placed to disclose HIV status, 22% thought that the primary caregiver was the best person, and more than half (56%) thought that it was best when both primary caregivers and healthcare workers were involved in disclosing HIV status to a child. When participants were asked about the rate of HIV disclosure at their facility, 51% reported that it was between zero and 25%. With regards to training on disclosure of HIV, less than half of the healthcare workers (47%) reported that they had received any in-service training.

### Barriers to HIV status disclosure

Table 2 presents the prevalence of healthcare workers who reported various barriers to HIV status disclosure. While more than four fifths of healthcare workers (more than 80%) identified inadequate knowledge of the HIV disclosure process, the lack of a standard tool for HIV disclosure, and the lack of training on HIV status disclosure as major barriers, almost two thirds (63%) also pointed to the unwillingness of primary caregivers to disclose as a reason for non-disclosure.

**Table 2:** Barriers to HIV status disclosure to children living with HIV (*N* = 168)

Characteristic	Strongly agree/ agree n (%)	Neither agree nor disagree n (%)	Strongly disagree/disagree n (%)
Inadequate knowledge on disclosure	142 (85)	10 (6)	16 (9)
Lack of standard tool for disclosure	141 (84)	9 (5)	18 (11)
Lack of training on disclosure	142 (85)	12 (7)	14 (8)
Pressure of work	79 (47)	26 (15)	63 (38)
Lack of cooperation with primary caregivers	74 (44)	17 (10)	77 (46)
Unwillingness of primary caregivers	106 (63)	11 (7)	51 (30)

### Socio-demographic characteristics associated with non-disclosure of HIV status to children

The socio-demographic characteristics of healthcare workers associated with non-disclosure of HIV status to children in both bivariate and multivariate statistics are presented in Table 3. In bivariate analysis, registered nurses were the professional group who were most likely never to disclose (67% non-disclosure). Registered nurses were five times [unadjusted odds ratio (uOR) 5.0; 95% confidence interval (CI): 1.7–14.3] more likely never to disclose than counsellors. The length of time spent working in an ART clinic was also associated with HIV non-disclosure. Those who had worked in a clinic for less than 2 years were three times more likely never to disclose (uOR 3.0; 95% CI:1.3–6.8) than those who had worked 2 years or more. Female healthcare workers were two times more likely than their male counterparts (uOR 1.9; 95% CI: 1.1–3.6) never to disclose. Healthcare workers who had no training in HIV disclosure were seven times (uOR 7.2; 95% CI 3.4–10.1) more likely never to disclose than those who had undergone training.

**Table 3:** Healthcare workers’ characteristics associated with non-disclosure of HIV status to children (*N* = 168)

Variable	Disclosed n (%)	Not Disclosed n (%)	uOR (95% CI)	aOR (95% CI)
Region				
South	50 (69)	31 (31)	1.0	
Central	33 (59)	15 (41)	1.5 (0.6–3.7)	
North	23 (62)	16 (38)	1.4 (0.6–2.9)	
Age				
21–30	36 (60)	24 (40)	1.8 (0.8–3.9)	
31–40	33 (58)	24 (42)	1.9 (0.9–4.3)	
> 40 years	37 (72)	14 (28)	1.0	
Profession				
Counsellor	35 (71)	14 (29)	1.0	1.0
Clinical officer	27 (69)	12 (31)	1.1 (0.4–2.9)	0.9 (0.3–2.7)
Nurse Technician	36 (64)	20 (36)	1.4 (0.6–3.2)	0.9 (0.3–2.3)
Registered Nurse	8 (33)	16 (67)	5.0 (1.7–14.3)**	2.6 (0.8–8.8)
Years of working in ART clinic				
0–1 year	17 (42)	23 (58)	3.0 (1.3–6.8)*	1.4 (0.5–3.6)
2 to 3 years	47 (70)	20 (30)	0.9 (0.4–2.0)	0.7 (0.3–1.6)
≥ 4 years	42 (69)	19 (31)	1.0	1.0
Gender				
Female	48 (56)	38 (44)	1.9 (1.1–3.6)*	2.4 (1.1–5.5)*
Male	58 (71)	24 (29)	1.0	
Received in-service disclosure training				
Yes	39 (44)	50 (56)	1.0	1.0
No	67 (85)	12 (15)	7.2 (3.4–10.1)***	7.7 (3.4–10.7)***

*uOR* unadjusted odds ratio, *aOR* adjusted odds ratio

*p < 0.05; **p < 0.01; ***p < 0.001

When factors that were significant at bivariate level were entered into the multivariate model, the odds of non-disclosure of HIV status were higher among female healthcare workers [adjusted odds ratio (aOR) 2.4; 95% CI: 1.1–5.5] and those who had not received any training in disclosure of HIV status (aOR 7.7; 95% CI: 3.4–10.7). Odds ratios for professional group and years spent working in an ART clinic were both attenuated and not statistically significant in the multivariate model.

## Discussion

Almost all healthcare workers in our study reported that it was important to disclose HIV status, with more than half stating that disclosure should be a shared responsibility between healthcare workers and primary caregivers. With regard to the research questions: 1. a significant proportion of healthcare workers reported that they had never disclosed HIV status to a child and only a quarter or less of the children at their facility had had their HIV status disclosed to them; 2. The main barriers to disclosure cited by healthcare workers were: inadequate knowledge on HIV disclosure, the lack of a standard tool for disclosure, the lack of training on disclosure, and the unwillingness of primary caregivers to disclose; and 3. Female gender and the lack of training on disclosure were independently associated with never having disclosed HIV status.

Consistent with our results, a recent South African study of 206 healthcare workers found that 89% felt it was important to disclose HIV to a child living with HIV [22]. Similar findings were also reported in a study conducted in Ethiopia [42]. There is evidence that healthcare workers support disclosure because of its benefits, such as helping the child to understand about their condition [43–45], improved drug adherence [22, 39, 42, 45], protecting the child from re-infection or others from the infection [22, 33], and empowering the child to take responsibility for their own health [40].

The most appropriate age to disclose HIV status to children remains contentious in sub-Saharan African countries despite the widely disseminated WHO guidelines [46]. Sixty-four per cent of healthcare workers in a recent South African study identified 11 to 14 years as the best age for HIV disclosure [31], while the majority of participants in this study and a recent study conducted in Tanzania recommended 6 to 12 years [47].

In contrast to our findings, the authors of a qualitative study conducted in Zimbabwe reported that the majority of healthcare workers and community leaders they surveyed indicated that healthcare workers were the best people to disclose HIV status to children [48]. On the other hand, two qualitative South African studies found that healthcare workers thought it was the primary caregiver’s responsibility to disclose to their child and that the healthcare workers’ responsibility was mainly to assist the primary caregiver with information and to provide emotional support to the child [22, 38]. The authors of another recent South African study reported that counsellors and nurses considered that their role was to provide psychosocial support rather than to disclose to the child, while doctors deemed that it was principally doctor’s role to disclose to the child [23].

The variability in findings regarding healthcare workers’ views about the appropriate age of disclosure and person to disclose HIV to a child in sub-Saharan Africa countries may be attributed to lack of a standardised disclosure protocol [33, 39, 42, 45]. This suggests that the WHO [46] guidelines published in 2011 for the disclosure of HIV status to children are not accessible to many healthcare workers in the region. In the absence of disclosure protocols, healthcare workers rely on their personal experience and clinical judgement to determine the appropriate age and person to disclose.

Consistent with the findings of the current study, healthcare workers in Kenya and Zimbabwe have reported that it is best for them to work with primary caregivers in disclosing HIV status to children [39, 40]. The authors of a South African study have commented that primary caregivers have the right to decide whether to disclose to their child or not, and if they choose to disclose, they have the right to make decisions regarding when, how, where and who is the best person to disclose [38]. However, in reality, the majority of primary caregivers do not disclose to their child because of concerns about the adverse effects of HIV disclosure and lack of knowledge and skills [49, 50]. Others believe that healthcare workers are the most appropriate people to coordinate disclosure for children because HIV is a focus of their practice [51], and they have knowledge and technical skills that primary caregivers lack [46]. They can use their therapeutic communication skills to help parents disclose to their children [52]. Continuous interaction between healthcare workers and the primary caregivers of children living with HIV has been shown to facilitate children’s acceptance of the condition, as well as improve their resilience [52, 53].

Although the majority of healthcare workers in the current study indicated that disclosure should be a joint activity between themselves and primary caregivers, close to two-thirds reported the unwillingness of primary caregivers as a barrier to disclosure. Recent research in Malawi has shown that parents are unwilling to have a conversation about HIV with their child as it is not culturally appropriate to talk to children about sexual issues [54], they are concerned that discussing HIV with their child might have a negative impact on the child’s wellbeing [54, 55], and stigma and discrimination directed at parents and children living with HIV are still common [55]. The implementation of policies that discourage stigma and discrimination [56], and encourage HIV literacy, in families and communities [57] will inspire healthcare workers to disclose HIV status to children. In addition, the study findings highlight the need for healthcare workers to recognise the challenges primary caregivers face regarding disclosure. In the authors’ experience, in Malawi, the nurse – patient relationship is more likely to be hierarchical where the nurse tells the patient what to do and the patient does as he or she is told, rather than empowering him or her in relationship that is more equal and supportive. While psychology and sociology are taught in nursing degree programs in Malawi, many nurses continue to be influenced by community sanctioned patterns of social behaviour and, perhaps, most importantly they are too busy providing medications and other essential medical care. This hierarchical model of care is very likely to be practised by medical doctors and other healthcare workers as well, with counsellors being the only healthcare group who are providing ongoing emotional support, despite their limited training [58].

This scenario is not unique to the care of children with HIV in sub-Saharan African countries. Quite some time ago in the United States, healthcare workers were implored to view children with HIV and their families as a unit of treatment and to appreciate their cultural background instead of adhering to the hierarchical model of care [59]. Another suggestion is for healthcare workers to assess cultural, religious, psychosocial, family, as well as community factors that can affect the disclosure of HIV to children [43]. Continuous communication between healthcare workers and caregivers can provide an enabling environment on which preparation for disclosure can be made possible [44]. Once the challenges related to disclosure have been identified, healthcare workers are in a better position to work with caregivers to find possible solutions and develop age appropriate disclosure plans [60, 61].

Given that the prevalence of disclosure reported by primary caregivers in sub-Saharan African countries ranges from 11 to 34% [15–20]. It is not surprising that a significant proportion of healthcare workers in this study reported that they had never disclosed HIV status to a child and that the rate of HIV disclosure at their facility was also very low. Similar to primary caregivers, healthcare workers in this, and other studies, have reported barriers to their disclosure of HIV to children. More than three-quarters of healthcare workers participating in a study conducted in South Africa reported that they had not received any formal training in disclosure and they lacked resources, such as practice manuals and printed materials for families [22]. A lack of training and disclosure resources was also reported as hindering healthcare workers’ involvement in HIV disclosure in qualitative studies conducted in Tanzania, Ethiopia, and Uganda [33, 39, 42, 45].

Most of the barriers to HIV disclosure identified by healthcare workers were associated with a perceived lack of preparation and support. More than 80% of healthcare workers who participated in the current study indicated that they lacked adequate knowledge about how to disclose HIV status to children. Less than half of the participants working in ART clinics had received any training on HIV disclosure. It is not surprising that the healthcare workers who had not received training on disclosure were almost eight times more likely not to disclose compared to those who had. We anticipate that widespread implementation of training for healthcare workers would lead to a significant increase in the rate of HIV disclosure to children in Malawi, however, training is not the only requirement for effective disclosure. The lack of standardised disclosure materials, such as manuals and printed items, also contributes to significant inconsistencies in the way disclosure is conducted in Malawi. Comprehensive training and standardised disclosure materials would not only help healthcare workers to have knowledge and confidence in supporting primary caregivers with the disclosure process, but they would be better equipped to provide emotional care to children living with HIV.

With regard to the gender difference, a study conducted in Zimbabwe also reported that male healthcare workers were more likely to disclose to children compared to their female counterparts [48]. In most African cultures a man is considered to be the head of his family and is given responsibility to make critical decisions regarding the health of family members [62, 63]. Women customarily take a passive role in such matters leaving men to make the important decisions. As disclosure of HIV to children is perceived to be a delicate issue, female healthcare workers might believe that it is not their role. On the other hand, male healthcare workers might feel obligated to disclose because of the responsibility they assume in African cultures for making decisions. Sociological literature shows that healthcare institutions not only reflect, but also maintain, societal norms and values [63]. In addition, literature shows that female healthcare workers are generally more empathetic in their provision of care to patients than males [63]. On average, the female healthcare workers in our study might have chosen not to disclose because they were more concerned with the negative impact of HIV disclosure on the child than their male counterparts.

Our study is the first to be conducted in Malawi to assess healthcare workers’ perspectives about HIV disclosure to children and also the first of its type in sub-Saharan Africa to estimate the proportion of healthcare workers who do disclose. While this study has many strengths, it is not without limitations. First, the exclusion of healthcare workers from health centres and private hospitals has limited the generalisability of the findings to all healthcare workers in Malawi. However, staff working in health centres were excluded because they see a very small proportion of children living with HIV, and the fact that the centres do not have specialised HIV clinics and are situated in remote localities. Staff working in private hospitals were excluded because, again, they see a small proportion of children. We speculate that rates of disclosure are even lower in the private hospital setting due to the fact that children’s parents who tend to have greater wealth, are more likely to want to hide the diagnosis to avoid the stigma associated with HIV. A second limitation that is common to all cross sectional studies is that causality cannot be attributed to the associations found.

## Conclusion

This study highlights the need for providing appropriate training in HIV disclosure for healthcare workers and the provision of standardised disclosure materials. While we recognise the need for further research regarding healthcare workers’ involvement in disclosure of HIV status to children, we are of the opinion that it is time to develop interventions that maximise the likelihood that all children aged between 6 and 12 years will gradually learn about their HIV status, as recommended by WHO. With this goal in mind, we have already undertaken the formative evaluation of a proposed intervention that will involve the distribution of a series of age-appropriate, culturally acceptable story books for children living with HIV in Malawi. We envisage that the story books and related disclosure materials will greatly assist healthcare workers to engage with primary caregivers in the disclosure process.

## Additional file

Additional file 1: Study questionnaire. The study questionnaire contains questions about participant socio-demographic information and current practice and challenges related to HIV disclosure to children. (DOCX 24 kb)

## Abbreviations

aOR: Adjusted odds ratio
ART: Antiretroviral
CI: Confidence interval
HIV: Human Immunodeficiency Virus
uOR: Unadjusted odds ratio
WHO: World Health Organization
