# Supplementary material for: Healthcare workers’ perspectives and practices regarding the disclosure of HIV status to children in Malawi: a cross-sectional study
Source: BMC Health Serv Res. 2018 Jul 11;18:540. doi: 10.1186/s12913-018-3354-9 (PMC6042360; doi:10.1186/s12913-018-3354-9)
Supplement: Supplementary file 1 — Study questionnaire. The study questionnaire contains questions about participant socio-demographic information and current practice and challenges related to HIV disclosure to children. (DOCX 24 kb) [file 12913_2018_3354_MOESM1_ESM.docx]

**Additional file 1**

**Study questionnaire**

**Study Title:** Healthcare workers’ perspectives and practices regarding the disclosure of HIV status to children in Malawi: A cross-sectional study.

The questionnaire will take less than 30 minutes to complete. Please respond to all questions with honesty, completeness and accuracy. You are free to withdraw from participating in this study at any time without penalty. All answers you give will be confidential. Please use the pen to complete the questionnaire.

Tick the box that corresponds to your answer and write clearly where required. For example:

What is your gender?

Female ☐

Male ☐


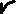


If your answer is male, then tick the box next to male with a pen as indicated above.

Name of health facility:

**PART A: DISCLOSURE OF HIV STATUS TO CHILDREN**

| **A1.** | **In your view, is it necessary to disclose HIV status to Children living with HIV?** | |
| --- | --- | --- |
|  | Yes ☐ | No ☐ |
|  | | |
| **A2.** | **Have you ever disclosed HIV status to a child?** | |
|  | Yes ☐ | No ☐ (Go to Question B6) |
|  | | |
| **A3.** | **Did you discuss with the child, HIV related issues such as causes, transmission and treatment, prior to disclosure of HIV status?** | |
|  | Yes ☐ | No ☐ |
|  | | |
| **A4.** | **Did you provide follow-up emotional support to the child after disclosure of HIV status?** | |
|  | Yes ☐ | No ☐ |
|  | | |
| **A5.** | **How would you describe the disclosure process?** | |
|  | Happened on one occasion only ☐ | Happened on 5-6 occasions ☐ |
|  | Happened on 2-3 occasions ☐ | Gradual process over 6 or more occasions ☐ |
|  | | |
| **A6.** | **In your view, at what age should a child be disclosed his/her HIV status?** | |
|  | Below 6 years ☐ | above 13 years ☐ |
|  | 6-12 years ☐ | I don’t know ☐ |
|  | | |
| **A7.** | **Who do you think is the best person to disclose HIV status to the child?** | |
|  | Primary caregiver ☐ | Teacher ☐ |
|  | Healthcare worker ☐ | Church minister ☐ |
|  | Primary care giver and healthcare worker ☐ | Community leaders ☐ |
|  | Other (please specify) ……………………………………………………………………………………… | |
|  | | |
| **A8.** | **At this facility, how would you rate the proportion of HIV disclosure to children living with HIV?** | |
|  | 0%☐ | 26-35 per cent ☐ |
|  | 5-10 %☐ | 36-45 per cent ☐ |
|  | 11-15 per cent ☐ | 46-55 per cent ☐ |
|  | 16-20 per cent ☐ | Above 55 per cent ☐ |
|  | 21-25 per cent ☐ |  |
|  | | |
| **A9.** | **Have you ever received in-service training on disclosure of HIV status to children?** | |
|  | Yes ☐ | No ☐ |

| **A10.** | **The following statements are factors that are known to hinder some healthcare workers from disclosing HIV status to children. For each statement, please indicate at present time if you would strongly disagree, disagree, neither agree nor disagree, agree or strongly agree (*Please circle* *appropriate number).*** | | | | | |
| --- | --- | --- | --- | --- | --- | --- |
|  |  | **Strongly disagree** | **disagree** | **Neither agree or disagree** | **Agree** | **Strongly agree** |
|  | Inadequate knowledge on how to disclose HIV status to children | 1 | 2 | 3 | 4 | 5 |
|  | Lack of a standard tool to use in disclosing HIV status to children | 1 | 2 | 3 | 4 | 5 |
|  | Lack of training for healthcare workers on disclosure of HIV status to children | 1 | 2 | 3 | 4 | 5 |
|  | Pressure of work | 1 | 2 | 3 | 4 | 5 |
|  | Lack of cooperation between healthcare workers and primary guardians | 1 | 2 | 3 | 4 | 5 |
|  | Unwillingness of the primary guardian to disclose | 1 | 2 | 3 | 4 | 5 |
|  | Other (please specify) ……………………………………………………………………………………... | | | | | |

**PART B: ABOUT YOU**

| **B1.** | **What is your age range?** | |
| --- | --- | --- |
|  | 21-25 years ☐ | 41 to 45 years ☐ |
|  | 26-30 years ☐ | 46-50 years ☐ |
|  | 31-35 years ☐ | Over 50 years ☐ |
|  | 36-40 years ☐ |  |
|  | | |
| **B2.** | **What is your gender?** | |
|  | Male ☐ | Female ☐ |
|  | | |
| **B3** | **What is your professional status?** | |
|  | Nurse technician ☐ | Counsellor ☐ |
|  | Registered nurse ☐ | Medical assistant ☐ |
|  | Clinical officer ☐ | Physician ☐ |
|  | Health surveillance assistant ☐ |  |
|  | Other (please specify) ……………………………………………………………………………………… | |
|  | | |
| **B4.** | **For how long you have worked in the antiretroviral therapy clinic?** | |
|  | Less than 6 months ☐ | 4 to 5 years ☐ |
|  | 6 months to one year ☐ | More than 5 years ☐ |
|  | 2 to 3 years ☐ |  |

Is there anything you would like to add?

………………………………………………………………………………………………………………………………………………………………………………………………………………………………………………....................................

End of questionnaire
